# Cardiovascular Disease Self-Care Interventions

**DOI:** 10.1155/2013/407608

**Published:** 2013-10-03

**Authors:** Victoria Vaughan Dickson, Jill Nocella, Hye-Won Yoon, Marilyn Hammer, Gail D'Eramo Melkus, Deborah Chyun

**Affiliations:** ^1^College of Nursing, New York University, 726 Broadway, 10th Floor, New York, NY 10003, USA; ^2^Department of Nursing, William Paterson University, Wayne, NY 07470, USA

## Abstract

*Background*. Cardiovascular disease (CVD) is a major cause of increased morbidity and mortality globally. Clinical practice guidelines recommend that individuals with CVD are routinely instructed to engage in self-care including diet restrictions, medication adherence, and symptom monitoring. *Objectives*. To describe the nature of nurse-led CVD self-care interventions, identify limitations in current nurse-led CVD self-care interventions, and make recommendations for addressing them in future research. *Design*. Integrative review of nurse-led CVD self-care intervention studies from PubMed, MEDLINE, ISI Web of Science, and CINAHL. Primary studies (*n* = 34) that met the inclusion criteria of nurse-led RCT or quasiexperimental CVD self-care intervention studies (years 2000 to 2012) were retained and appraised. Quality of the review was assured by having at least two reviewers screen and extract all data. *Results*. A variety of self-care intervention strategies were studied among the male (57%) and Caucasian (67%) dominated samples. Combined interventions were common, and quality of life was the most frequent outcome evaluated. Effectiveness of interventions was inconclusive, and in general results were not sustained over time. *Conclusions*. Research is needed to develop and test tailored and inclusive CVD self-care interventions. Attention to rigorous study designs and methods including consistent outcomes and measurement is essential.

## 1. Introduction 

Cardiovascular disease (CVD) is a major cause of morbidity and mortality worldwide [1]. It is estimated that 1 in 3 American adults have CVD. After age of 40, the lifetime risk of developing CVD is 49% for men and 32% for women [2]. Although advances in medical and surgical management of CVD have substantially reduced cardiac mortality rates in the United States (US), individuals with CVD remain at increased risk for further cardiac events, including unstable angina, myocardial infarction, and heart failure [1]. Cardiovascular disease in the US costs more than $108 billion each year [3], which includes the cost of health care services, medications, and lost productivity. 

Individuals with CVD are routinely instructed to engage in self-care behaviors as part of daily disease management. Numerous terms are used interchangeably with self-care including self-management, self-regulation, self-monitoring, adherence, and compliance to describe the behaviors or activities in which patients are asked to engage in to promote health and well-being [4]. In the cardiovascular literature, self-care refers to adherence to treatment recommendations, symptom response, and adoption of healthy lifestyles like smoking cessation and weight management [5]. Education aimed at promoting these self-care behaviors is incorporated into in all major clinical practice guidelines for CVD [6].

Self-care is a fundamental concern for nursing and a nursing research priority. In fact, the National Institute of Nursing Research (NINR) strategic plan [7] emphasizes patients' self-management of chronic illness symptoms and treatment. To that end, there has been an increase in research efforts that seek to evaluate strategies that help people live with chronic illness and maintain or improve their quality of life, develop self-management strategies to increase support systems and improve the patient's and the family's understanding of the chronic illness, and focus on coping with symptoms associated with chronic illness.

Generally, self-care interventions take place in several ways: on a one-to-one basis between the patient and health care provider; in disease-specific group education programs; in settings including clinical locations or at home; delivered by either peer leaders or health providers; and through interactive technology [8]. According to NINR, the primary goal of self-care including self-management interventions is to improve health and quality of life outcomes in patients with chronic conditions [7]. One way that interventions are hypothesized to be effective is by empowering patients to increase their understanding of their condition and take responsibility for their health; increasing self-efficacy is another common mechanism [8]. Researches targeting specific chronic conditions (e.g., diabetes, cancer, arthritis, HIV/AIDS) have found that self-care interventions are associated with condition-specific, patient-centered outcomes like improved glycemic control [9, 10], better pain control [11], improved sleep [12], and better functional status [13]. Less is known about the effects of self-care interventions on economic outcomes such as healthcare utilization in these conditions. Research to identify effective strategies are essential to developing evidence-based recommendations that can be translated into clinical practice.

Although self-care of chronic conditions has been a nursing research priority for over a decade, recent improvements in CVD outcomes have accelerated the need to develop and test CVD self-care interventions that improve patient-centered outcomes. In 2009, the American Heart Association (AHA) published a scientific statement on self-care as integral to management of heart failure [14], which has been echoed in the 2013 guidelines from the interdisciplinary American College of Cardiology Foundation/American Heart Association Task Force [15]. These recommendations as well as other CVD practice guidelines [6] provide suggestions for what should be included in self-care interventions. Although there has been an increase in the number of self-care studies, there remains a lack of clarity on the impact of CVD self-care interventions. To date, few CVD self-care interventions have been adopted as evidence-based practice. 

Therefore, the purpose of this integrative review was to describe the nature of nurse-led CVD self-care interventions. Specifically, we answer 3 questions: (1) what are the CVD self-care intervention strategies and how are they deployed?, (2) what populations are targeted?, and (3) what are the outcomes studied in CVD self-care interventions? We also identify limitations in current nurse-led CVD self-care interventions and make recommendations for addressing them in future research. An integrative review approach was appropriate for this analysis because it allowed for the inclusion of diverse methodologies, specifically varied intervention approaches, as well as inclusion of a range of CVD diagnoses in order to generate a comprehensive description of the “nature” of nurse-led CVD self-care interventions [16].

## 2. Methods 

### 2.1. Eligibility Criteria

Cardiovascular disease (CVD) was defined as disorders of the heart and blood vessels [1, 17] inclusive of coronary heart disease, cerebral vascular disease, peripheral vascular disease, heart failure, arrhythmias, and heart valve disease. Consistent with the conceptual definition of self-care as a set of behaviors or activities that patients are asked to engage in to promote health and well-being [4], interventions that focused on self-care including adherence, compliance, self-care maintenance, self-care management, symptom monitoring, and self-management were selected. Since self-care is a fundamental concern of nursing and focus of increased research efforts [7], only nurse-led studies defined as studies conducted by a nurse primary investigator (PI) were included in this review. We acknowledge that there are many self-care interventions that include a nursing component or are directed by nurses. However, given the aims of this review, we limited the search to only those studies conducted by a nurse PI. 

The search was limited to the dates of 2000 through 2012 primarily because advances in CVD treatment have led to improved survival rates in the past decade [1] resulting in an increased emphasis on patient self-care after a cardiac event or illness. The search was restricted to intervention studies that were randomized controlled trials (RCTs) or quasiexperimental studies in which there was a control group. 

### 2.2. Information Sources

A comprehensive search of the literature was conducted using PubMed, MEDLINE, ISI Web of Science, and Cumulative Index of Nursing and Allied Health Literature (CINAHL). Hand searching of references was also conducted. 

### 2.3. Search

Search terms were selected based on definitions of CVD [1, 17] and self-care [16]. Search terms and strategies were developed in consultation with the research team who are experts in self-care research and with a medical librarian. The search strategy used the National Library of Medicine's Medical Subject Headings (MeSH) key word nomenclature. All related terms and combinations of terms related to self-care and CVD were used in the initial search. The literature search was then refined to identify intervention studies that were RCTs or quasiexperimental studies with a control group. Finally, the literature was reviewed and filtered to select studies with nurse as PI. 

### 2.4. Study Selection

Selected studies were limited to those with adult populations (age ≥ 19) with CVD diagnosis (“coronary heart disease,” “coronary artery disease,” “heart failure,” “cardiomyopathy,” “hypertension,” “cardiovascular disease,” “peripheral vascular disease,” “cerebral vascular disease,” “stroke,” “arrhythmia,” and “valve disease”). 

Only nurse-led self-care interventions were included in this review. Studies had to identify a self-care component to the intervention, for example, self-care, self-care maintenance, self-care management, adherence, symptom-monitoring, symptom management, and self-management. Nurse as PI was determined by (1) reference as PI status, (2) first author was nurse, or (3) senior author was nurse.

This review included RCTs and quasiexperimental studies. Only studies that reported original data and had a comparison or control group were included. 

After the initial search of the literature, each title and abstract were examined independently by two reviewers. Initially, 95% agreement on relevance was achieved. In cases where reviewers disagreed (5%), articles were discussed with the review team in order to gain consensus. All articles identified as relevant were then screened for eligibility by two reviewers and if criteria were met advanced to data abstraction. 

### 2.5. Data Collection Process

The data extraction process was conducted by 3 investigators. First, a data extraction form was created based on the aims of the review and piloted on the first 3 studies by 2 of the investigators. Data were compared and confirmed by team members, and data extraction form was refined. Subsequently all studies under went a dual review for data abstraction (i.e., 2 of 3 investigators reviewed each article). In this way, quality measures used throughout the process of screening through data abstraction supported protection against bias and enhanced consistency and accuracy of findings reported in this review. 

### 2.6. Data Abstraction Process

Abstracted data elements included first and last author name and discipline, discipline of PI if designated, country of study, purpose, study design, sample characteristics (CVD diagnosis, gender, age, ethnicity/race), sample size, theoretical framework, intervention (type, description), measurement timeframe, main study outcomes, reported outcomes/results, stated key findings, stated or reviewer observed limitations, and attrition rate (number and reason, if reported).

### 2.7. Synthesis of Results

Data were summarized across studies to describe the nature of nurse-led interventions including the type of intervention (content, mode of delivery, dose, frequency, and theory-based), population studied (gender, and race), methods (randomization process, instruments, psychometrics), outcomes (measurement intervals and results), and limitations; and then by CVD diagnosis. Then data were analyzed to identify common limitations and generate recommendations for future research.

## 3. Results

### 3.1. Study Selection and Characteristics

**Figure 1:**
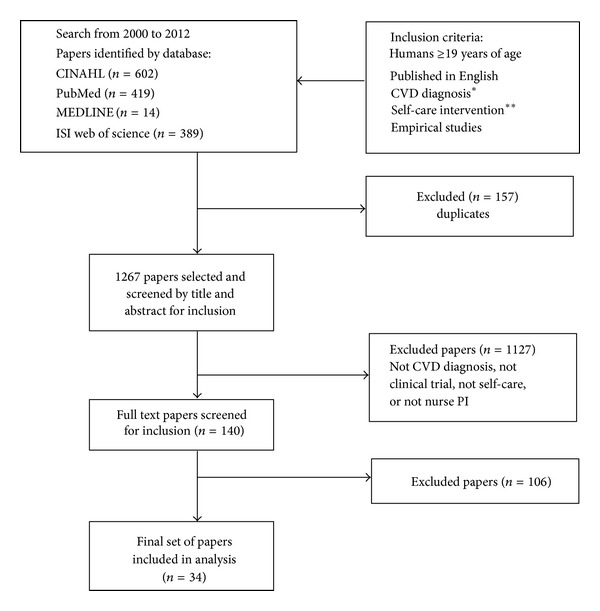
PRISMA flowchart. * “cardiovascular disease,” “coronary heart disease,” “coronary artery disease,” “heart disease,” “congestive heart failure,” “heart failure,” “hypertension,” “cerebral vascular disease,” “stroke,” “peripheral vascular disease,” “vascular disease,” “arrhythmia.” ** “self-care,” “self-management,” “self-care maintenance,” “self-care management,” “symptom management,” “symptom monitoring,” “adherence,” “compliance” AND “intervention” OR “education.”

The search initially produced 1424 studies; 34 met the inclusion criteria (Figure 1) and were analyzed (Table 1). Of these 34 studies, 24 were from USA, 10 studies were international studies, and 1 study was a multicenter international study (i.e., Australia and USA); 30 were RCTs and 4 were quasiexperimental studies. The majority (*n* = 23) focused on heart failure diagnosis, 8 targeted coronary heart disease and/or acute coronary syndrome, and 3 examined interventions for persons with other CVD conditions—arrhythmia, hypertension, and vascular disease. 

### 3.2. Synthesis of Results 

*Question Number 1*. *What Are the CVD Self-Care Intervention Strategies and How Are They Deployed?* There were a myriad of strategies described in this literature including individualized interventions in which the content was tailored to the needs of the patient or behaviorally focused, structured education, telemonitoring intended to support self-care behaviors (e.g., medication reminders, blood pressure checks), and disease management that integrated case management, monitoring, and education. Most of the studies in this review (18 of 34) were combined interventions and consisted of multiple strategies, including combinations of education, behavioral component, and individualized care through multiple modalities (e.g., in-person and telephone follow-up), or were part of a disease management approach (*n* = 4). 

The delivery method of interventions included telephonic [22, 24, 25, 39], multimedia/computer [31, 36, 42, 44], group based [10], and in-person (one-on-one) [31, 35–38, 40, 45, 51].

In addition, the setting, in which interventions were conducted varied and included in-hospital or predischarge after a cardiac event [41], outpatient or clinical setting and in-home. Commonly, interventions were initiated in the hospital or clinical setting with follow-up contacts in the home environment. This approach leveraged hospital resources to facilitate transition from hospital to home [28], a vulnerable point in CVD self-care, or augmented existing services like home health care with innovative interventions [20, 21, 32]. 

Intervention lengths ranged from 3 days to 17 months (mean 14 weeks SD 16.12 weeks, median 8 weeks). The frequency of intervention contact varied and was not reported in several of the studies, making it difficult to assess dose. 

Seventeen of the 34 studies described a theoretical framework or conceptual model, either nursing or behavioral, as guiding the development, implementation, or evaluation of the intervention. Five studies were guided by nursing theories: (1) Rogers' science of unitary human being [42], (2) Orem's self-care deficit theory [19, 22], and (3) Riegel's self-care of heart failure conceptual model [32, 39]. However, the most commonly used conceptual framework used was Bandura's cognitive social theory and theory of self-efficacy [10, 25, 26, 44, 45]. Other behavioral theories used were the health belief model [41], transtheoretical model of stages of change [37], health promotion model [51], and theory of self-regulation [27]. The importance of a theoretical framework to clearly describe the theoretical relationships and measurement of self-care is highlighted by Jaarsma et al. who examined the effects of a theoretically derived supportive educational nursing intervention on self-care abilities, self-care behaviors, and quality of life in patients with HF [30]. Their results that self-care only contributed partially to quality of life indicated that in some populations a more intensive self-care intervention is needed. That is, self-care interventions need to be tailored as to content and dose in order to be effective.

*Question Number 2*. *What Are the Populations Targeted?* As noted, heart failure was the most common CVD diagnosis addressed by the self-care interventions. Across the 34 studies reviewed, pooled demographic statistics show 57% male and 67% Caucasian. It is important to note that 19 studies did not report race. Only 2 studies focused on ethnic minority populations [10, 39]. Lorig and colleagues evaluated the health and utilization outcomes of a 6-week community-based peer-led program for Spanish speakers with heart disease [10]. At 4 months, the intervention group (*n* = 327), as compared with usual-care control subjects (*n* = 224), demonstrated improved health status, health behavior, and self-efficacy, as well as fewer emergency room visits (*P* < 0.05). At 1 year, the improvements were maintained and remained significantly different from baseline status. 

Riegel et al. examined the effectiveness of telephonic disease management that included a focused self-care intervention in decreasing hospitalizations and improving health-related quality of life (HRQL) and depression in Hispanics of Mexican origin with HF [39]. Although they used bilingual nurses to adapt the intervention, there were no significant group differences in HF hospitalizations, the primary outcome variable (usual care: 0.49 ± 0.81 (CI 0.25–0.73); intervention: 0.55 ± 1.1 (CI 0.32–0.78) at 6 months), or other outcomes of HF readmission rate, HF days in the hospital, HF cost of care, all-cause hospitalizations or cost, mortality, HRQL, or depression. Collectively, the results from these two rigorously designed and conducted studies stress the importance of ensuring adequate diversity in sample populations and continued research to address the unique needs of ethnically diverse populations.

Unfortunately, the proportion of ethnic minorities represented in other studies of this review was very small and subgroup analysis was not performed by any of the studies. 

*Question Number 3*. *What Are the Outcomes Studied in CVD Self-Care Interventions?* The most common outcomes reported in this literature were quality of life, reported by 19 studies while healthcare utilization outcomes including emergency room use, hospital days, were studied in 12 studies. Measurement of these outcomes varied across studies; for example, there were 9 different quality of life measures used including general quality of life measures (e.g., Medical Outcome Study Short Form-36 [52]) and condition specific measures (e.g., Minnesota Living with Heart Failure [53], MacNew Heart Disease Health-related Quality of Life [54]). Interestingly, few (*n* = 10) reported a self-care result; yet measures of self-care either objective or subjective were reported in 16 of the 34 studies. Measurement of physical as well as psychosocial outcomes varied widely throughout the studies. Cardiac-related outcomes were measured by the 6-minute walk test (*n* = 5), blood pressure (*n* = 3), cholesterol (*n* = 2), and B-Natriuretic Peptide (BNP) levels (*n* = 4). Mood (i.e., depression and anxiety) was measured (*n* = 9) using 7 different scales. Most studies measured outcomes at multiple intervals, commonly at 3–6 months. 

### 3.3. Limitations of Current Nurse-Led CVD Self-Care Interventions

This integrative review highlighted three overarching limitations in the current nurse-led CVD self-care intervention research: (1) lack of sample diversity, (2) inconclusive results within studies, and (3) methodological weaknesses in study design. 

#### 3.3.1. Lack of Sample Diversity

As noted earlier, the studies in this review were predominately male and Caucasian; only 2 studies focused on ethnic minority populations [10, 39]. The lack of sample diversity is a significant limitation and demonstrates the continued need for increased participation in research by women and ethnic minority populations, who continue to experience poorer CVD outcomes [1].

#### 3.3.2. Inconclusive Results within Studies

Only 11 studies reported statistically significant between-group improvement in at least one primary outcome measured; 13 studies reported improvement in one or more primary outcome in the intervention group but not between groups. Only 3 studies reported sustained positive results over time [10, 35, 47]. Inconclusive findings are a significant limitation in that they confuse interpretation of results and impedes the translation of relevant findings into practice. 

There are several potential explanations for inconclusive findings: lack of self-care measurement; inadequate measurement of outcomes; and combined interventions that make it difficult to parcel out the effective intervention component. First, although all of the studies in this review were self-care interventions, self-care was only measured in 16 of the studies. Therefore, studies that did not measure self-care were limited in their ability to link the intervention to the primary outcome, which may have contributed to mixed findings within a single study. 

Use of subjective measures also confounded the results even in well-designed RCTs. For example, Prasun et al. (2005) tested a self-directed diuretic titration intervention compared to usual care in a sample of 66 adults with HF [38] and measured physiological outcomes (i.e., B-Natriuretic Peptide), behavioral outcomes, and healthcare utilization and mortality at baseline and at 3 months. There was a significant difference between groups in healthcare utilization and exercise capacity. The intervention group who self-titrated diuretics better (60% compared to 40% in control group) had fewer self-reported HF-related emergency visits (2.8% [1] versus 22.7% [7], *P* = 0.15) compared to the usual care group and improved significantly in exercise capacity (646 ± 60 ft versus 761 ± 61 ft, *P* = 0.01) measured by the 6-minute walk test. Since ER visits are common in HF patients and mostly due to symptom exacerbation of fluid overload [55], these results suggest that a diuretic titration intervention may be feasible in promoting self-care, specifically symptom management. Although assessment of physiological markers of fluid overload and myocardial stress [56], along with the 6-minute walk test, are significant strengths of this study, researchers relied on self-report of HF-related healthcare utilization without verification by medical records, which weakens results. It is also not clear if those in the usual care group were instructed to use the ER as the venue for diuretic titration, which could introduce bias into the study and contribute to the inconclusive results within the study.

Also, many studies reported combined interventions making it difficult to ascertain the effective component of an intervention which was a limitation when findings were inconclusive. Brandon et al. reported positive outcomes including improved hospital readmissions, quality of life and self-care behaviors when comparing intervention group who received the advanced practice nurse-led telephonic enhanced disease management and self-care education to the usual care group [22]. Self-care behaviors were measured by the Self-Care Behavior scale and improved significantly in the intervention group compared to the usual care group (*F*(1,18) = 21.8, *P* = 0.001) thereby linking the self-care outcome to the specific intervention component that focused on self-care adherence (e.g., medication). However, it was less clear if the effect on the primary outcomes of interest (hospital readmission decreased in intervention group (*F*[1,18] = 7.63, *P* = 0.013) and improvement in quality of life (*F*[1,18] = 5.80, *P* = 0.026) can be attributed to the self-care intervention or perhaps the clinical care or disease management delivered by the physician and nurse, respectively. 

#### 3.3.3. Methodological Weaknesses

There were several common methodological weaknesses found in this integrative review that may also help explain the equivocal results. A number of the studies were pilot studies and/or had small sample sizes [19–21, 23, 33, 37]; thus they were underpowered to detect potentially important differences. Many studies used inappropriate statistical techniques to assess changes over time, using pairwise comparisons between groups at each timepoint or comparing within group changes. Several studies did appropriately use survival analyses when looking at time to first event between groups [22, 24, 25, 48]: analysis of covariance [10] or mixed methods modeling [32, 33]/repeated measures analysis of variance [20, 21, 33, 35, 38, 41, 46] to detect changes interaction effects of time by group changes. 

Weak fidelity of treatment monitoring was another methodological weakness. Few studies described a method whereby they monitored or documented the delivery of the intervention. An example of gold standard in treatment fidelity was use of objective assessment via tape-recording of the intervention adherence to a protocol [43]. Other less objective methods included self-appraisal and observer assessment [23, 35, 49]. Studies vaguely described usual care as “standard care” delivered by physician, nurse, or variety of healthcare providers [18] or a control treatment that was similar to the intervention intended to control for attention effect. Therefore, usual care may have differed among those allocated to the control group. In these cases, fidelity monitoring would have identified variance in usual care and perhaps helped explain findings. 

## 4. Discussion

### 4.1. Summary of Evidence

The purpose of this integrative review was to describe the nature of nurse-led CVD self-care interventions and identify limitations of this literature in order to generate recommendations for future research. We found that a range of strategies including a variety of modes of delivery have been tested in this population with varying results. We found a glaring lack of subject diversity in this body of research. This finding is of particular concern because cardiovascular disease is a leading cause of morbidity and mortality worldwide and ethnic minority groups experience disproportionate burden and poorer outcomes, as do women. In addition, inconclusive results and combined interventions make it difficult to identify effective program attributes or pose recommendations for clinical use based on the current findings. Further, we found methodological weaknesses in many of the studies included in this review that threaten both external validity (i.e., small sample sizes skew results and decrease ability to generalize findings of the study) and internal validity (i.e., selection bias, attrition, and combined intervention decrease ability to make an inference that the independent variable is truly influencing the dependent variable). 

In the following section, the limitations in our review and how we minimized these challenges are discussed. Then implications for future research that includes recommendations for addressing the current limitations in nurse-led CVD self-care intervention research are presented.

### 4.2. Limitations of the Review

There are several limitations to this review. As described above, the studies in this review included RCTs and quasiexperimental and varied methodological approaches that preempted ability to conduct any meta-analyses. There was variation in how studies reported ethnicity/race which affected our pooled results of demographics. Numerous instruments were used to study common outcomes (e.g., quality of life) without consistency across interventions or outcomes. Therefore, it was difficult to compare results across studies especially when psychometrics were not reported. Further, the lack of description about the intervention and control group treatments was a significant limitation in reporting the results of this analysis. It may be that the lack of clarity in the descriptions resulted in miscategorization of the study intervention in this analysis. Statistical methods in the analysis of several studies were often either not adequately described or not appropriate, which may have contributed to nonsignificant results as well as influenced our assessment of study. We addressed these challenges by following a rigorous review process in which each study was reviewed by at least two investigators. Statistical methods for each included study were also reviewed independently by an expert on our team. Definitions for categorization of type of intervention were developed and used during data abstraction. Ambiguity in studies was discussed by the entire team until a consensus was reached and in the case where interventions were inadequately described referenced materials were reviewed (e.g., methods papers describing the intervention). 

A second limitation in our integrative review may be our *a priori* decision to define nurse-led CVD self-care intervention studies as those in which the PI was a nurse. Our purpose was expressly to describe the nature of nurse-led interventions, and therefore we included only studies where the PI was a nurse rather than studies led by other disciplines with a nurse as a research team member. It is possible that our search may have missed studies where a nurse was PI but not credited as such in the paper nor listed as the first or last author of the study. We made every effort to identify the discipline of the PI by checking funding sources where PI and discipline would be identified, checking academic and department affiliation, and contacting authors. Interestingly, we did not find any cost-effective analyses or comparative effective studies in this review. It may be that by excluding studies where the nurse was not the PI, these studies were missed in this review. 

Finally, since the lines between self-care interventions and other CVD patient education interventions sometimes can be unclear [4], we may have missed interventions that had a self-care component. We minimized this limitation by conducting a rigorous search with quality-monitoring in each phase that included careful review of the description of each intervention prior to inclusion. 

### 4.3. Recommendations for Future Research Based on Findings

The 34 studies examined in this review represent a significant body of CVD self-care intervention research conducted over the past 10 years. The results of this integrative review are important because they highlight ongoing limitations in this area and inform recommendations to address the gaps in future CVD self-care intervention research. 

Unfortunately, our results regarding the lack of sample diversity are not new [57–60]; but they highlight the need for renewed focus on recruitment strategies to enroll an adequate representation of women and minorities as well as retention strategies to minimize attrition [61]. Such efforts should include outreach to communities and community leaders to facilitate engagement of ethnic minority populations and incorporate culturally appropriate interventions [62, 63]. In addition, strategies to reduce attrition need to be integrated into study design up front [61].

Addressing the significant limitation of lack of sample diversity in future research is paramount and has implications for overcoming health disparities in the CVD population. In 2012, the Department of Health and Human Services developed a formal *Action Plan to Reduce Racial and Ethnic Health Disparities* [64] that placed emphasis on the conduct of health disparities research. A key part of the action plan is to target patient-centered outcomes research among racial and ethnic minority populations; CVD was a priority area. The national initiative, in conjunction with Healthy People 2020, aims to achieve health equity and eliminate disparities such as those that exist in CVD for subpopulations (i.e., race, ethnicity, and gender). Our results suggest more work is needed in the areas of adequate representation of women and minorities in research and culturally appropriate CVD self-care interventions. 

Future research must also employ rigorous study design and methods in order to establish effectiveness of interventions for translation into clinical practice [58, 65, 66]. Recommendations to address the common methodological weaknesses include enlisting an interdisciplinary team of experts led by a nurse scientist. Collaborating with a statistician as well as experts in content area to strengthen initial study design stage may help overcome some of the common methodological weaknesses [66] like inadequate power or statistical methods and fidelity monitoring. Further, consistent use of reliable and precise measures such as those included in the Patient Reported Outcomes Measurement Information System (PROMIS) toolbox [67] would facilitate integration and assessment of effectiveness of CVD self-care intervention research in the future. Consistent measurement will also facilitate collaboration among nurse scientists working in similar programs of research and help move this science forward. 

Results of this integrative review suggest that incorporation of a theoretical framework may strengthen CVD self-care intervention research [30], a finding advocated by others [57, 68]. Self-care is a fundamental nursing phenomenon, the focus of nursing theorists and a nursing sensitive outcome identified by the American Academy of Nursing. Use of theoretical frameworks has utility in CVD self-care intervention research by delineating factors to address in an intervention as well as linking self-care to desired outcomes [69]. In clinical practice, a theoretically derived intervention can help nurses identify individuals vulnerable to poor self-care and guide a plan of care that incorporates self-care. 

Finally, consistent with other reviews [66, 68, 70, 71], we found that the use of combined interventions was very common and led to questions about variance in dose of intervention as well as content. For example, Chodosh et al.'s meta-analysis of 53 chronic disease self-management studies (including 19 hypertension studies) concluded that interventions “probably” were beneficial but the elements of the programs that were effective could not be determined [71]. That is, what is it about a combined intervention that makes it effective? Research is needed that rigorously tests the structure, process, and outcomes of an intervention in order to identify the mechanism of effectiveness [66, 72]. In complex combined interventions, evaluation should include fidelity monitoring, calculation of intervention dose, and precise outcome measurement. Qualitative methods can help identify the mechanism of effectiveness and support treatment fidelity especially when interventions are “tailored” [73]. 

## 5. Conclusions

This integrative review identified significant shortcomings in the existing nurse-led CVD self-care intervention research. Research is needed to develop and test tailored and inclusive CVD self-care interventions that are guided by an appropriate theoretical framework. Attention to rigorous study designs and methods is critical. This review reinforces the continued importance of adequate representation in CVD self-care intervention research by diverse populations and the need to develop and test culturally appropriate interventions. As the number of patients with CVD continues to increase worldwide, improving self-care in this population takes on added importance. Nursing research has a critical role to play in advancing the science of CVD self-care. 

## Figures and Tables

**Table 1:** Description of nurse-led CVD self-care interventions.

Study and location	Sample (as reported)	Intervention/control	Primary outcomes and measurement	Key findings
Albert et al. (2007); USA [18]	*N* = 112 Gender: males *n* = 86 Ethnicity: Caucasian *n* = 93 CVD diagnosis: HF Attrition not reported	IG: multimedia (video education) CG: standard education by physician and/or nurse	*Healthcare resource utilization*: hospitalization, emergency care, office visits, and laboratory tests medical records *Self-care/adherence*: adapted from SCHFI *Functional class*: NYHA status change *Timeframe*: baseline, 3 m	(1) 3-month healthcare utilization (*P* = NS) (2) IG had greater sign/symptom recognition (*P* < .04) and higher mean self-care behavior/adherence (*P* < .01)

Artinian et al. (2003); USA [19]	*N* = 18 Gender: males *n* = 17 Ethnicity: Black *n* = 11 Caucasian *n* = 6 CVD diagnosis: HF Attrition not reported	IG: web-based monitoring CG: usual care	*Self-care*: HFSCBS *Medication adherence*: pill counts *QOL*: MLHF *Timeframes*: baseline, 3 months	(1) Improved QOL in IG (*F* = 10.0, *P* = .006), (*P* = .002); not CG (*P* = .113) (2) Better adherence in IG versus CG (*P* = NS)

Barnason et al. (2006); USA [20]	*N* = total 50 Gender: males *n* = 28 Ethnicity not reported CVD diagnosis: CHD Attrition not reported	IG: combined intervention of telemonitoring and home visit CG: usual care	*QOL*: SF-36 *Healthcare utilization*: emergency care *Timeframe*: baseline, 6 weeks, 3 m	(1) IG had higher QOL general health functioning (*F* = 8.41, *P *< .01) (2) Significant time effects in QOL physical (*F* = 9.42, *P *< .01), role-physical functioning (*F* = 5.74, *P *< .05) in both groups (3) CG had more ER visits (NS)

Barnason et al. (2009); USA [21]	*N* = 55 Gender: males *n* = 46 Ethnicity: White *n* = 54, nonwhite *n* = 1 CVD diagnosis: CHD Attrition not reported	IG: telehealth intervention CG: usual care	*QOL*: SF-36 *Physical activity*/*energy expenditure*: RT3 accelerometer *Timeframe*: baseline, 3 w, 6 w, 3 m, 6 m	(1) Significant main effect by group in energy expenditure/physical activity (*F* = 4.66, *P *< .05) (2) Both groups had significantly improved QOL *P *< .05)

Brandon et al. (2009); USA [22]	*N* = 20 Gender: males = 9 Ethnicity: Caucasian *n* = 8, African American *n* = 12 CVD diagnosis: HF Attrition not reported	IG: nurse-led telephone intervention (7 telephone calls, 5–30 minutes in length) CG: usual care with standard education by physician and/or nurse	*QOL*: MLHF *Self-care*: Self-Care Behavior scale *Healthcare utilization*: self-report hospitalizations *Timeframe*: baseline, 3 m	(1) IG improved self-care behaviors (*F* = 21.853, *P *< .001) and reduced hospital readmissions (*F* = 7.63, *P* = .013) (2) QOL in IG improved *P* = NS; no change in UC

Caldwell et al. (2005); USA [23]	*N* = 36 Gender: males *n* = 25 Ethnicity: white *n* = 34, other = 2 CVD diagnosis: HF Attrition *n* = 11	IG: combined intervention: focused education and counseling with telephone follow-up CG: usual care	*Self-care*: EHFScBS *Biomarkers*: BNP *Timeframe*: baseline, 3 m	(1) Self-care improved significantly in IG (*P* = .03) (2) No significant difference in BNP levels (*P* = .21)

DeBusk et al. (2004); USA [24]	*N* = 462 Gender: males *n* = 236 Ethnicity: white *n* = 386, Black *n* = 27, Hispanic *n* = 14, American Indian *n* = 27, Asian *n* = 8 CVD diagnosis: HF Attrition *n* = 72	IG: telephonic case management CG: usual care	*Healthcare utilization*: HF and all-cause hospitalizations medical claims *Timeframe*: baseline, 12 m	(1) HF rehospitalization similar in both groups (NS) (proportional hazard, 0.85 (95% CI = 0.46, 1.57)) (2) All-cause rehospitalization NS (proportional hazard, 0.98 (95% CI = 0.76, 1.27))

Dougherty et al. (2005); USA [25]	*N* = 168 Gender: males *n* = 139 Ethnicity: Caucasian *n* = 150, American Indian/Alaska *n* = 3, Asian/Pacific Islander *n* = 4 CVD diagnosis: arrhythmia Attrition *n* = 18	IG: combined intervention: self-care management patient education, telephone, and clinical support CG: usual care	*QOL*: SF-36 *Depression*: CES-D *Healthcare utilization*: outpatient visits, hospitalizations, and emergency care *Timeframe*: baseline, 6 m, 12 m	(1) Improved mood in IG (*P* = .04) compared to CG (2) No statistically significant differences between the groups on total outpatient visits, hospitalizations, or ER visits over 12 months

Gallagher et al. (2003); Australia [26]	*N* = 196 Gender: 196 females Ethnicity not reported CVD diagnosis: CHD Attrition not reported	IG: combined intervention: telephone intervention with behavioral focus CG: usual care	*Depression*: Hospital Anxiety and Depression Scale *Timeframe*: baseline, 12 w	(1) No significant differences in anxiety (*F* = 0.15, *P* = .69) or depression (*F* = 0.11, *P* = .74) between groups

Gould (2011); USA [27]	*N* = 154 Gender not reported Ethnicity not reported CVD diagnosis: CHD Attrition *n* = 25	IG: combined intervention: discharge nursing intervention with telephone follow up (IG, *n* = 64) CG: usual care	*Adherence*: Morisky adherence *Healthcare utilization*: urgent care *Timeframe*: baseline, 3 days	(1) No significant group differences were found on medication adherence, or use of urgent care

Harrison et al. (2002); Canada [28]	*N* = 192 Gender: males *n* = 105 Ethnicity not reported CVD diagnosis: HF Attrition *n* = 8	IG: combined intervention: transition/discharge care: educational materials, telephone, and home visits CG: usual care included home visits	*QOL*: MLHF, SF-36 *Healthcare utilization*: emergency care, readmission rates (medical records) *Timeframe*: baseline, 6 weeks, 12 weeks	(1) IG: improvement in QOL (27.2 ± 19.1) compared to the CG (37.5 ± 20.3; *P* = .002) (2) Less emergency room use in transitions group compared to CG (*P* = .03) but no change in readmission rates

Holmes-Rovner et al. (2008); USA [29]	*N* = 525 Gender: males *n* = 191 Ethnicity: Non-Hispanic white *n*-443, African American *n* = 60, Hispanic White *n* = 12 CVD diagnosis: CHD/acute coronary syndrome Attrition *n* = 152	IG: telephonic intervention with behavioral focus CG: usual care	*Functional status/physical activity*: Duke Activity Status Index BP *Timeframe*: baseline, 3 m, 8 m	(1) IG showed higher physical activity (OR = 1.53, *P* = .01) during the first three months (2) No significant differences in functional status or QOL

Jaarsma et al. (2000); the Netherlands [30]	*N* = 179 Gender: males *n* = 79 Ethnicity not reported CVD diagnosis: HF Attrition *n* = 47	IG: combined intervention of education, telephone, and home visits (6 encounters) CG: usual care	*Self-Care*: HFSCBS *QOL*: Cantril's Ladder *Timeframe*: baseline, 1 m, 3 m, 9 m	(1) Self-care behaviors improved in IG (1 m (*t* = 3.3, *P *< .001), 3 m (*t* = 2.9, *P* < .005) but not sustained at 9 m (*t* = 0.7, *P* = .47)) (2) QOL in both groups at 3 m not sustained at 9 m (3) Limited effect of self-care on QOL (*r* = 0.24, *P *< .05)

Kutzleb and Reiner (2006); USA [31]	*N* = 23 Gender: males = 8 Ethnicity not reported CVD diagnosis: HF Attrition not reported	IG: combined intervention: individualized education and counseling with telephone follow-up CG: usual care: protocol driven medical care	*QOL*: Ferrans and Powers QOL Index Functional status: 6-minute walk test *Timeframe*: baseline, 12 m	(1) IG: improved QOL (*F* = 3.569, *P <*.000) (2) IG: functional capacity NS (*F* = 0.228, *P* = .949) (3) Between-group NS

LaFramboise et al. (2003); USA [32]	*N* = 90 Gender: males = 45 Ethnicity: Caucasian *n* = 75, African American *n* = 12, other *n* = 3 CVD diagnosis: HF Attrition not reported	Combined intervention Group 1: Telephonic only Group 2: Home visit only Group 3: Telemonitoring Group 4: Home visit and Telemonitoring *All groups also received structured HF disease management 5 encounters	*Functional status*: 6-minute walk test *Self-efficacy*: BEES-HF *Depression*: Geriatric Depression Scale *QOL*: SF-36 *Timeframe*: baseline, 2 m:	(1) Group by time effect significant (*P* = .0027) in self-efficacy only (2) Improved functional status (*P* < .01), HRQL (*P* < .05), and depression (NS) in all groups

Lorig et al. (2003); USA [10]	*N* = 551 Gender: males *n* = 113 Ethnicity: U.S. born *n* = 31 Mexican born *n* = 353, Central American born *n* = 121, South American born *n* = 36 CVD diagnosis: CHD Attrition not reported	IG: group-based, peer-led community-based program CG: usual care	*Healthcare utilization*: emergency care, hospitalizations, *Timeframe*: baseline, 6 w, 4 m, 12 m	(1) IG had fewer emergency room visits (*P* < .05) at 4 m and 1 year (*P *< .001)

Maric et al. (2010); Canada [33]	*N* = 20 Gender: males *n* = 11 Ethnicity not reported CVD diagnosis: HF Attrition *n* = 3	IG: combined intervention: web-based education and monitoring with telephone follow-up CG: usual care	*Self-care*: SCHFI *Functional status*: 6-minute walk test *Biomarkers*-BNP *Timeframe*: baseline, 6 m	(1) Improved self-care (*P* = .039) (3) no change in QOL, (*P* = .337), 6-minute walk test (*P* = .124), and BNP (*P* = .210)

Mårtensson et al. (2005); Sweden [34]	*N* = 153 Gender: males *n* = 83 Ethnicity not reported CVD diagnosis: HF Attrition not reported	IG: combined intervention: individualized education and counseling with telephone follow up CG: usual care	*QOL*: MLHF, SF-36 *Depression*: Zung self-rated depression scale *Timeframe*: baseline, 3 m	(1) No significant difference in QOL; but IG preserved QOL while UC deteriorated in QOL (*P* = .035), vitality (*P* = .029) (2) No significant differences in depression

McKinley et al. (2008); Australia and USA [35]	*N* = 3522 Gender: males *n* = 2,393 Ethnicity: white *n* = 3,207, other *n* = 315 CVD diagnosis: CHD Attrition *n* = 386	IG: combined intervention: individual one-on-one education provided with structured education with counseling CG: usual care	*Mood*: Multiple Affect Adjective Check List *Timeframe*: baseline, 3 m, 12 m	(1) Knowledge increased significantly from baseline in IG compared to CG at 3 months and sustained at 12 months (*P* = .0005 for all) (2) Higher state anxiety was associated with lower levels of knowledge (*P *< .05)

Otsu and Moriyama (2009); Japan [36]	*N* = 96 Gender: males *n* = 61 Ethnicity not reported (Japanese study) CVD diagnosis: HF Attrition *n* = 3	IG: individualized (face-to-face) case management CG: usual care	*QOL*: Macnew health-related quality of life *Functional status*: NYHA *Biomarkers*; BNP *Mortality*: records *Timeframe*: baseline, 3 m, 6 m, 9 m, 12 m	(1) Statistically significant differences between groups: BNP at 3 m (*P* = .032) and 6 m (*P* = .002) (2) IG: improved QOL in IG improved (*F* = 26.157, *P *< .000) (3) No significant difference in NYHA but deterioration in symptom in the UC group (NS)

Paradis et al. (2010); Canada [37]	*N* = 30 Gender: males *n* = 22 Ethnicity not reported CVD diagnosis: HF Attrition *n* = 5	IG: combined intervention: motivational interview (3 encounters—1 in person; 2 telephone) CG: usual care	*Self-care*: SCHFI *Timeframe*: baseline, 1 m	(1) No significant results in self-care behaviors (2) IG: improved self-care confidence (*P* = .005)

Prasun et al. (2005); USA [38]	*N* = 66 Gender: males *n* = 43 4) Ethnicity: white *n* = 58, African American *n* = 7, other *n* = 1 CVD diagnosis: HF Attrition not reported	IG: supportive education about flexible diuretic titration CG: usual care	*QOL*: MLHF *Functional status*: 6-minute walk test *Biomarkers*: BNP, norepinephrine *Healthcare utilization*: emergency care, hospitalizations, mortality *Timeframe*: baseline, 3 m	(1) IG: improved 6-minute walk test (646 ± 60 ft versus 761 ± 61 ft, *P* = .01) and total QOL score (53 ± 5 versus 38 ± 5, *P* = .001), no change in CG group (2) Significantly fewer emergency care in the IG compared to CG (3% versus 23%, *P* = .015) (3) No differences in hospitalizations or mortality (4) No differences were found between baseline and 3-month biomarkers

Riegel et al. (2006); USA [39]	*N* = 134 Gender: males *n* = 62 Ethnicity: Hispanics *n* = 134 (109 Spanish-speaking) CVD diagnosis: HF	IG: telephonic case management with self-care education CG: usual care	*Self-care*: SCHFI *Depression*: Patient Health Questionnaire-9 *Healthcare utilization*: hospitalizations, cost, mortality—medical records *Timeframe*: baseline, 3 m, 6 m	(1) No significant group differences were found in HF readmission rate, HF days in the hospital, HF cost of care, all-cause hospitalizations or cost, mortality, or depression

Scott et al. (2004); USA [40]	*N* = 88 Gender: males *n* = 39 Ethnicity not reported CVD diagnosis: HF Attrition *n* = 22	Group 1: individualized counseling and usual care Group 2: supportive-educative and usual care Group 3: usual care and placebo	*QOL*: SF-36 *Depression*: Mental Health Inventory *Timeframe*: baseline, 6 m	(1) IG (groups 1 and 2) improved QOL (*F* = 4.632, *P* = .01) and depression (*F* = 6.27, *P* = .003) and over a 6-month period (2) between-group comparisons (NS)

Sethares and Elliott (2004); USA [41]	*N* = 70 Gender: males *n* = 33 Ethnicity: white *n* = 63, black *n* = 6 CVD diagnosis: HF Attrition *n* = 18	IG: combined intervention, individualized/tailored message intervention CG: usual care	*QOL*: MLHF *Healthcare utilization*: hospitalizations *Timeframe*: baseline, 1 w, 1 m	(1) No significant differences in HF readmission rates or QOL

Shearer (2007); USA [42]	*N* = 90 Gender: males *n* = 56 Ethnicity: white *n* = 81, black *n*-2, Hispanic *n* = 3, Native American *n* = 1 CVD diagnosis: HF Attrition *n* = 3	IG: telephonic intervention with behavioral focus CG: usual care	*Self-care*: self-management heart failure *QOL*: SF-36 *Timeframe*: baseline, 3 m	(1) IG improved self-care compared to CG (*F* = 6.19, *P *< .001) (2) QOL NS

Shively et al. (2005); USA [43]	*N* = 116, Gender: males *n* = 110 Ethnicity: Caucasian *n* = 87, African American *n* = 11, Hispanic *n* = 9, Asian/Pacific Islander *n* = 6, mixed *n* = 3 CVD diagnosis: HF Attrition = 15	IG: combined intervention: behavioral management with telephone follow up CG: usual care	*QOL*: SF-36, MLHF *Functional status*/exercise capacity: 6-minute walk test *Timeframe*: baseline, 4 m, 10 m, 16 m	(1) IG improved QOL compared to UG (*F* = 7.04, *P* = .009) (2) No group differences in exercise capacity

Smeulders et al. (2010); the Netherlands [44]	*N* = 317 Gender: males *n* = 230 Ethnicity not reported CVD diagnosis: HF Attrition *n* = 42	IG: group-based structured education CG: usual care	*Self-care*: EHFScBS *QOL*: SF36, Kansas City Cardiomyopathy Questionnaire *Depression*: HADS *Timeframe*: baseline, 26 weeks, 52 weeks	(1) IG improved in self-care (*P* < .01) and QOL (*P* = .005) (2) results not sustained at 6 and 12 months

Sol et al. (2010); the Netherlands [45]	*N* = 314 Gender: male *n* = 242 Ethnicity not reported CVD diagnosis: vascular disease Attrition *n* = 91	IG: tailored behavioral self-care intervention CG: usual care	*QOL*: SF-36 *Biomarkers*: lipids, BP, waist circumference, BMI *Timeframe*: baseline, 1 yr	(1) IG achieved treatment goals for LDL-cholesterol (difference 13%, 95% CI = 1, 26) and HDL-cholesterol (difference 9%, 95% CI = 0, 19) compared to CG (2) Mean SBP decreased significantly by 5 mm Hg (95% CI = −9, 0) in IG (3) BMI increased significantly by 0.4 kg/m^2^ (95% CI = −0.8, −0.1) in CG (4) No significant differences were seen in waist circumference, smoking, or triglycerides or QOL

Stafford and Berra (2007); USA [50]	*N* = 419 Gender not reported Ethnicity not reported CVD diagnosis: CHD Attrition *n* = 122	IG: combined intervention: individualized case management with follow-up meetings, telephone call, home visits CG: primary care	Framingham risk score *Timeframe*: baseline, 17 m	(1) IG had statistically significant reduction in mean Framingham risk probability compared to CG (1.6% decrease in 10-year CHD risk, *P* = .007)

Strömberg et al. (2003); Sweden [47]	*N* = 106 Gender: males *n* = 65 Ethnicity not reported CVD diagnosis: HF Attrition *n* = 43	IG: combined intervention: group based intervention focused on self-care education and support to patient and family CG: usual care	*Self-care*: HFSCBS *Healthcare utilization*: hospitalizations, length of stay, mortality *Timeframe*: baseline, 12 m	(1) IG: fewer patients with events (death or admission) after 12 months compared to CG (29 versus 40, *P* = .03) and fewer deaths after 12 months (7 versus 20, *P* = .005) (2) IG had fewer admissions (33 versus 56, *P* = .047) and days in hospital (350 versus 592, *P* = .045) during the first 3 months (3) At 12 months, there was a 55% decrease in admissions/patient/month (0.18 versus 0.40, *P* = .06) and fewer days in hospital/patient/month (1.4 versus 3.9, *P* = .02) (4) IG improved in self-care at 3 and 12 months compared to CG (*P* = .02 and *P* = .01)

Strömberg et al. (2006); Sweden [48]	*N* = 154 Gender: males *n* = 109 Ethnicity not reported CVD diagnosis: HF Attrition *n* = 24	IG: multimedia intervention CG: usual care	*QOL*: EuroQol *Adherence*: study-specific survey *Timeframe*: baseline, 1 m, 6 m	(1) NS difference between groups in adherence or QOL

Tonstad et al. (2007); Norway [49]	*N* = 51 Gender: males *n* = 36 Ethnicity not reported CVD diagnosis: hypertension Attrition *n* = 4	IG: combined intervention: behavioral intervention with telephone follow up focuses on lifestyle counseling CG: primary care	*Biomarkers*: lipids, triglycerides BP, Waist circumference *Timeframe*: baseline, 6 m	(1) Waist circumference increased significantly between baseline and 6 m in CG but not in IG (mean difference 3.1 cm (95% CI 1.2–5.0), *P* = .04) (2) Reduced serum triglyceride in IG compared with CG (mean difference 0.56 mmol/L (95% CI 0.22–0.90), *P* = .03)

Westlake et al. (2007); USA [46]	*N* = 80 Gender: males *n* = 57 Ethnicity: white *n* = 58, black *n* = 8, Hispanic *n* = 3 other *n* = 11 CVD diagnosis: HF Attrition not reported	IG: web-based education (*n* = 40) CG: standard education	*QOL*: SF-36 *Timeframe*: baseline, 3 m	(1) Between-group improvement in QOL (*P *< .001)

BMI: body mass index; BNP: B-Natriuretic Peptide; BP: blood pressure; CG: control group; CHD: coronary heart disease; CVD: cardiovascular disease; EHFScBS: European Heart Failure Self-Care Behavior Scale; HF: heart failure; HFSCBS: Heart Failure Self-Care Behavior Scale IG: intervention group; MLHF: Minnesota Living with Heart Failure Questionnaire; NS: not significant; NYHA: New York Heart Association; QOL: quality of life; SCHFI: Self-Care of Heart Failure Index.
